# A Model-Based Assessment of the Seizure Onset Zone Predictive Power to Inform the Epileptogenic Zone

**DOI:** 10.3389/fncom.2019.00025

**Published:** 2019-04-26

**Authors:** Marinho A. Lopes, Marc Goodfellow, John R. Terry

**Affiliations:** ^1^Living Systems Institute, University of Exeter, Exeter, United Kingdom; ^2^Wellcome Trust Centre for Biomedical Modelling and Analysis, University of Exeter, Exeter, United Kingdom; ^3^EPSRC Centre for Predictive Modelling in Healthcare, University of Exeter, Exeter, United Kingdom

**Keywords:** epilepsy surgery, ictogenic network, seizure onset zone, epileptogenic zone, neural mass model

## Abstract

Epilepsy surgery is a clinical procedure that aims to remove the brain tissue responsible for the emergence of seizures, the epileptogenic zone (EZ). It is preceded by an evaluation to determine the brain tissue that must be resected. The identification of the seizure onset zone (SOZ) from intracranial EEG recordings stands as one of the key proxies for the EZ. In this study we used computational models of epilepsy to assess to what extent the SOZ may or may not represent the EZ. We considered a set of different synthetic networks (e.g., regular, small-world, random, and scale-free networks) to represent large-scale brain networks and a phenomenological network model of seizure generation. In the model, the SOZ was inferred from the seizure likelihood (SL), a measure of the propensity of single nodes to produce epileptiform dynamics, whilst a surgery corresponded to the removal of nodes and connections from the network. We used the concept of node ictogenicity (NI) to quantify the effectiveness of each node removal on reducing the network's propensity to generate seizures. This framework enabled us to systematically compare the SOZ and the seizure control achieved by each considered surgery. Specifically, we compared the distributions of SL and NI across different networks. We found that SL and NI were concordant when all nodes were similarly ictogenic, whereas when there was a small fraction of nodes with high NI, the SL was not specific at identifying these nodes. We further considered networks with heterogeneous node excitabilities, i.e., nodes with different susceptibilities of being engaged in seizure activity, to understand how such heterogeneity may affect the relationship between SL and NI. We found that while SL and NI are concordant when there is a small fraction of hyper-excitable nodes in a network that is otherwise homogeneous, they do diverge if the network is heterogeneous, such as in scale-free networks. We observe that SL is highly dependent on node excitabilities, whilst the effect of surgical resections as revealed by NI is mostly determined by network structure. Together our results suggest that the SOZ is not always a good marker of the EZ.

## Introduction

Epilepsy is one of the most common neurological disorders affecting approximately 50 million people worldwide. Anti-epilepsy drugs are the first line of treatment for epilepsy and they provide sufficient seizure control in around two thirds of cases (Kwan and Brodie, 2000). Surgery is an option for the remaining individuals who are not seizure-free under medication. Epilepsy surgery is preceded by a qualitative assessment of different brain imaging modalities in order to try to identify the brain tissue responsible for the generation of seizures, the *epileptogenic zone* (EZ) (Rosenow and Lüders, 2001). Surgeons can only be sure that the EZ was correctly inferred if as a result of it being removed through surgery the patient has become seizure-free (Rosenow and Lüders, 2001). Intracranial electroencephalography (iEEG) is commonly used during the presurgical assessment to find the seizure onset zone (SOZ) (David et al., 2011; Duncan et al., 2016) which is assumed to be a marker of the EZ (Rosenow and Lüders, 2001). In fact, the SOZ may or may not be a good proxy of the EZ depending on whether it is concordant with other data modalities (Duncan et al., 2016). The assumption underlying the use of the SOZ is that the region where seizures emerge, the *seizure focus*, is at least part of the brain tissue responsible for the seizures. However, the EZ and the SOZ may not coincide (Rosenow and Lüders, 2001), which may explain why epilepsy surgery is often unsuccessful and long-term positive outcome may be lower than 25% in extra-temporal cases (de Tisi et al., 2011; Najm et al., 2013). The question arises as to whether the SOZ is a suitable marker of the EZ.

Computational models have recently been proposed to quantitatively examine clinical data and determine targets for surgery (Hutchings et al., 2015; Goodfellow et al., 2016, 2017; Khambhati et al., 2016; Lopes et al., 2017, 2018; Sinha et al., 2017). These methods use MRI or iEEG data acquired during presurgical workup to infer structural or functional brain networks. The rationale to assess brain networks is based on recent advances in our understanding of epilepsy that indicate that seizures may arise from distributed ictogenic networks (Richardson, 2012; Bartolomei et al., 2017; Besson et al., 2017). We and others have shown that brain networks may be evaluated, and relevant information extracted by placing a mathematical model of seizure activity into each node of the network and examine network dynamics (Hutchings et al., 2015; Goodfellow et al., 2016; Lopes et al., 2017; Sinha et al., 2017). In this context, two different approaches have been used to predict targets for surgery. In refs. (Hutchings et al., 2015; Sinha et al., 2017), the authors mimicked the clinical presurgical procedure by simulating the SOZ, assuming this is a proxy for the EZ. They used a phenomenological model of seizure transitions to compute the escape time, i.e., the time that each network node takes to transition from a normal state to a seizure-like state. Nodes with the lowest escape time were considered representative of the SOZ and therefore candidates for surgical resection. In contrast, in our studies we simulated different possible surgeries *in silico* with the aim of evaluating how effective each one was at stopping seizures (Goodfellow et al., 2016; Lopes et al., 2017). In particular, surgeries were modeled by removing a node from the network and its effect was quantified by measuring the network propensity to generate seizures before and after node removal. Nodes whose removal led to seizure freedom *in silico* were defined to be targets for surgery. Both methods proved to be predictive of surgical outcome and hold promise to be used to optimize presurgical assessment of the EZ (Hutchings et al., 2015; Goodfellow et al., 2016, 2017; Lopes et al., 2017, 2018; Sinha et al., 2017).

Herein we are going to use this computational framework to examine how well the SOZ may or may not be a marker of the EZ *in silico*.

## Materials and Methods

### Computational Model

To assess whether the SOZ is a reliable proxy to infer targets for epilepsy surgery, we model surgery on large-scale brain networks, where each network node may produce seizure activity depending on both its internal excitability and interaction with other nodes. A node represents a portion of brain tissue which may be responsible for the emergence of seizures across the network, and surgery is modeled as node removal from the network.

We consider a phenomenological model of seizure dynamics, the theta model (Lopes et al., 2017, 2018). Each node dynamics is characterized by a phase θ_*i*_ described by the following first-order ordinary differential equation (ODE):

$$\begin{array}{l} {\overset{∙}{\theta }}_{i}=\left(1-\cos\theta_{i}\right)+\left(1+\cos\theta_{i}\right)I_{i}\left(t\;\right), \end{array}$$

where *I*_*i*_ (*t*) is the input current of node *i*. Depending on *I*_*i*_ (*t*), the node is either at a fixed stable phase $\theta_{i}^{\left(s\right)}$ (*I*_*i*_ < 0), or oscillating in a limit cycle (*I*_*i*_ > 0). The boundary *I*_*i*_ = 0 between the two states corresponds to a saddle-node on invariant circle (SNIC) bifurcation. This simple model has been shown to be a computationally efficient and a reliable approximation of a more complex and biophysical realistic model of epileptiform dynamics (Lopes et al., 2017). The phase $\theta_{i}^{\left(s\right)}$ represents a “normal state” of brain activity whereas the limit cycle corresponds to a “seizure state.”

Node excitability and interaction with other nodes are expressed in the current,

$$\begin{array}{l} I_{i}\left(t\right)=I_{0}^{\left(i\right)}+\xi_{i}\left(t\right)+\frac{K}{N}\underset{j\neq i}{\sum }a_{ji}\left[1-\cos\left(\theta_{j}-\theta_{j}^{\left(s\right)}\;\right)\right], \end{array}$$

where $I_{0}^{\left(i\right)}$ is the excitability of node *i*, ξ_*i*_(*t*) are noisy inputs, *K* is the global scaling coupling of network interaction, *a*_*ji*_ is *j*,*i*^th^ entry of the adjacency matrix that encodes the network, $\left[1-\cos\left(\theta_{j}-\theta_{j}^{\left(s\right)}\right)\right]$ defines the output of node *j*, and $\theta_{j}^{\left(s\right)}$ is its steady state. The noisy inputs represent signals from other areas of the brain outside of the network under consideration. The noise follows a Gaussian distribution with zero mean and variance σ^2^. Each node receives independent noise,

$$\begin{array}{l} \langle \xi_{i}\left(t\right)\xi_{j}\left(t^{\prime }\right)\rangle =\sigma^{2}\delta_{i,j}\delta \left(t-t^{\prime }\right). \end{array}$$

The steady state $\theta_{i}^{\left(s\right)}\;$is obtained from setting ${\overset{∙}{\theta }}_{i}=0$ in the absence of noise and inputs from other nodes (ξ^(*i*)^(*t*) = 0 and *K* = 0),

$$\begin{array}{l} \theta_{i}^{\left(s\right)}=-Re\{\cos^{-1}(\frac{1+I_{0}^{\left(i\right)}}{1-I_{0}^{\left(i\right)}})\;\}. \end{array}$$

At $I_{0}^{\left(i\right)}<0$, there are two fixed points, one stable ($\theta_{i}^{\left(s\right)}$), and one unstable (${-\theta }_{i}^{\left(s\right)}$). We take the real part so that θ^(*s*)^ = 0 at *I*_0_ > 0.

Following our previous study (Lopes et al., 2017), we use a fixed standard deviation σ = 0.6 for all nodes. The excitability *I*_0_ is set to −1.2, except when otherwise specified. The differential equation was integrated using the Euler-Maruyama method.

This framework enables us to study and characterize brain networks. A brain network may be inferred from different data modalities (Bullmore and Sporns, 2009) and can be represented by an adjacency matrix *a*_*ji*_. The mathematical model is then a means to understand how the network structure influences the potential emergence of seizures across the network.

### Seizure Likelihood

The SOZ is classically defined as the brain region from which clinical seizures are generated (Rosenow and Lüders, 2001). In network models of seizure dynamics, the SOZ can be defined in a similar fashion: the SOZ corresponds to the network nodes where seizure-like activity is first observed. When applied to real brain networks, the modeling is thus capable of suggesting whose nodes (i.e., brain regions) are more likely to be sources of seizure activity. In particular, Sinha et al. constructed functional networks from inter-ictal iEEG recordings and assessed them by finding whose network nodes would first spike in a phenomenological model of seizure transitions (Sinha et al., 2017). Model dynamics was quantified using the inverse of the escape time, the seizure likelihood (Sinha et al., 2017). We had previously introduced the escape time as the time that each network node takes to “escape” from normal activity to the seizure state (Benjamin et al., 2012; Petkov et al., 2014). Nodes that escape faster have a high seizure likelihood and correspond to the model-based SOZ.

Here we redefine the seizure likelihood measure as

$$\begin{array}{l} SL^{\left(i\right)}=\;\frac{1}{T}\int_{K_{1}}^{K_{2}}t_{sz}^{\left(i\right)}\left(K\right)dK\; \end{array}$$

where $t_{sz}^{\left(i\right)}\left(K\right)$ is the amount of time that node *i* spends in spiking dynamics during a sufficiently large reference time *T* (we use *T* = 4 × 10^6^ time steps). For a fixed global coupling *K*, *SL*^(*i*)^ corresponds to the fraction of time node *i* spends in seizure dynamics, i.e., the likelihood of finding the node in the seizure state. We take the integral over *K* in order to account for the dependence of $t_{sz}^{\left(i\right)}$ on *K*, avoiding an arbitrary choice of *K*. We choose a sufficiently large interval [*K*_1_, *K*_2_] to capture the full variation of $t_{sz}^{\left(i\right)}$ (as *K*→*K*_1_, $t_{sz}^{\left(i\right)}\to 0$, whereas when *K*→*K*_2_, $t_{sz}^{\left(i\right)}\to T$). Finally, we normalize *SL*^(*i*)^ by the maximum *SL* across the network, so that *SL* is confined to the range [0, 1]. Nodes with *SL*^(*i*)^ = 1 are the SOZ *in silico*, whereas nodes with *SL*^(*i*)^ = 0 do not participate in seizure activity. The average *SL* across all network nodes for a fixed *K* corresponds to the *Brain Network Ictogenicity* (*BNI*) (Chowdhury et al., 2014; Petkov et al., 2014; Goodfellow et al., 2016; Lopes et al., 2017) and is an advance on the integral redefinition of BNI introduced in Lopes et al. (2018). This redefinition of seizure likelihood is essentially equivalent to the measure used in Sinha et al. (2017): nodes that escape faster to seizures are the nodes that spend longer times in seizure dynamics. The reason to use the *SL* within the theta model instead of the measure based on the escape time is that the *SL* is computationally more efficient and robust.

### Node Ictogenicity

To quantify the result of epilepsy surgery, we have previously introduced the concept of *Node Ictogenicity* (*NI*) (Goodfellow et al., 2016; Lopes et al., 2017). *NI*^(*i*)^ measures the relative difference in *BNI* upon removing node *i* from a network:

$$\begin{array}{l} NI^{\left(i\right)}=\frac{BNI_{pre}-BNI_{post}^{\left(i\right)}}{BNI_{pre}} \end{array}$$

where *BNI*_*pre*_ is *BNI* prior to node resection, and $BNI_{post}^{\left(i\right)}$ is *BNI* after the removal of node *i*. As in previous works, we set parameters such that *BNI*_*pre*_ = 0.5 (Goodfellow et al., 2016; Lopes et al., 2017). $BNI_{post}^{\left(i\right)}$ is computed with the same parameters as *BNI*_*pre*_, and typically yields a value smaller than *BNI*_*pre*_. Some nodes may render the network free from spiking dynamics $BNI_{post}^{\left(i\right)}=0$ which corresponds to *NI*^(*i*)^ = 1, whist others may not affect *BNI* ($BNI_{post}^{\left(i\right)}=BNI_{pre}$, and thus *NI*^(*i*)^ = 0). In this framework the EZ corresponds to the nodes with highest *NI*. Whereas, *SL* is based solely on a presurgical network, using SOZ as a proxy for the EZ, *NI* quantifies the result of different possible surgical resections by comparing the dynamics of a post-surgical network with the presurgical network, therefore using *in silico* surgeries to define the EZ.

### Comparison Measures

We aim to examine whether *SL* is equivalent to *NI* to clarify whether the SOZ is concordant with the EZ *in silico*. Assuming that the SOZ is predictive of the pathological tissue that must be resected, then we would expect a high correlation between *SL* and *NI*. We use a weighted Kendall's rank correlation measure (Carterette, 2009; Kumar and Vassilvitskii, 2010; Lopes et al., 2017) to compare *SL* and *NI*. We calculate

$$\begin{array}{l} \tau =\frac{P-Q}{P+Q} \end{array}$$

where *P* is the number of groups of two nodes with the same order in the two rankings (*SL* and *NI*), and *Q* is the number of groups in reverse order. We take into account the relative values of *SL* and *NI* by weighting each contribution to *P* and *Q* by the product of the distances in *SL* and *NI*, |*SL*^(*i*)^ − *SL*^(*j*)^| × |*NI*^(*i*)^ − *NI*^(*j*)^|. τ = 1 means that *SL* and *NI* rankings have the same ordering, whist τ = −1 corresponds to the two rankings in reverse order.

The weighted Kendall's rank τ does not account for relative differences between *SL* and *NI* as long as the two rankings have the same order. For example, the sorted values of *SL* may grow linearly, whist the sorted values of *NI* may show an exponential growth. τ does not capture this difference. If the nodes are equally ordered by the two measures, then τ = 1 independently of the values of *SL* and *NI*. We thus also calculate the Pearson correlation ρ to compare *SL* and *NI* across network nodes. In turn ρ does not depend strongly on the ordering of the distributions and that is why we use both τ and ρ to perform the comparison between *SL* and *NI*.

### Network Topologies

We have previously shown that the *BNI* and *NI* depend on network topology (Lopes et al., 2017). Therefore, to compare the *SL* with *NI* it is important to consider different network topologies. We study regular, small-world, random, and scale-free networks, both directed and undirected (Watts and Strogatz, 1998; Goh et al., 2001; Newman, 2003; Lee et al., 2004; Miši et al., 2014). To construct a set of small-world networks we used the Watts-Strogatz algorithm (Watts and Strogatz, 1998) and 9 rewiring probabilities (*p* = 0.1, 0.2, …, 0.9). The small-world properties of these networks were confirmed using the small-world measure (Telesford et al., 2011). Note that for *p* = 0 we obtain regular networks, whereas for *p* = 1 the algorithm generates random networks (Watts and Strogatz, 1998). In the case of undirected scale-free networks, we used the static model (Goh et al., 2001) and examined 11 different topologies characterized by degree distributions $P_{k}\propto k^{-\alpha }$ with a range of exponents α = 2, 2.3, 2.6, …, 5. The smaller the exponent α the more heterogeneous the network is with respect to the number of connections per node. Directed scale-free networks were generated using Barabási-Albert algorithm (Albert and Barabási, 2002). All networks consisted of 64 nodes and for each network topology we studied three mean degrees *c* = 4, 8, 16. The reason to focus on networks with 64 nodes is that this is the typical size of networks inferred from intracranial EEG, where the number of electrodes may span from about 30–100 (Goodfellow et al., 2016). We discarded networks with disconnected components and considered 10 networks realizations per network topology. Therefore, we studied 1,010 networks in total (see Table 1).

**Table 1:** Studied networks. *p* is the rewiring probability to obtain small-world networks, and α is the degree distribution exponent of scale-free networks.

**Networks**		
**Topologies**	**Undirected**	**Directed**
Regular	10 n.r.	10 n.r.
Small-world	*p* = 0.1, 0.2, …, 0.9	*p* = 0.1, 0.2, …, 0.9
	10 n.r. per *p*	10 n.r. per *p*
Random	10 n.r.	10 n.r.
Scale-free	α = 2, 2.3, 2.6, …, 5 10 n.r. per α	α = 3 10 n.r.

*We considered 10 network realizations (n.r.) per topology. The table thus indicates 34 topologies and a total of 340 networks. We generated networks with three different mean degrees, c = 4, 8, 16 (in the case of directed networks, it corresponds to mean in- and out- degree). We did not consider scale-free networks with α = 2 and c = 4, as these did not verify the condition of no disconnected components. Therefore, we studied 340 × 3 −10 = 1010 networks*.

### Homogeneous and Heterogeneous Excitabilities

Network nodes may differ from each other due to both topological properties in the network, and their intrinsic excitabilities $I_{0}^{\left(i\right)}$. Nodes characterized by higher excitabilities are more susceptible to spontaneously generate spiking activity due to noisy inputs and also to be recruited into seizure dynamics by other nodes.

Previous studies have considered homogeneous excitability distributions, i.e., all nodes described by the same excitability *I*_0_ (Goodfellow et al., 2016; Lopes et al., 2017, 2018; Sinha et al., 2017). This assumption was used for simplicity and due to a lack of available data to make an informed decision about specific excitabilities in different brain regions. Although it remains unclear how to obtain these data, we can nevertheless study how heterogeneous excitabilities may impact on *SL* and *NI*. For comparison, first we consider networks with homogeneous excitabilities, and then two different cases with heterogeneous excitabilities. In the homogeneous case, all network nodes have the same excitability parameter $I_{0}^{\left(i\right)}=I_{0}$ and are completely equivalent except for their local network topological properties described in the adjacency matrix *a*_*ji*_.

Rummel et al. has studied iEEG data of individuals with refractory epilepsy and found a fraction of 8.3% of channels in the SOZ. Given that the SOZ may be the consequence of localized hyper-excitable brain tissue, we distinguish a group of six randomly chosen nodes (i.e., about 9% of the network) with higher excitabilities ($I_{0}^{\left(h\right)}$) compared to the other nodes with fixed *I*_0_ = −1.2. We consider the existence of these hyper-excitable nodes in regular, small-world, and random networks. Note that in these networks, we have previously shown that *NI* is mostly similar across nodes when excitabilities are homogenous (Lopes et al., 2017). Thus, here we aim to understand whether hyper-excitable nodes may or may not be detected by *SL* and *NI*. We studied 5 random selections of hyper excitable nodes per network and examined separately $I_{0}^{\left(h\right)}=-1$ and −0.1, i.e., weakly and strongly hyper-excitable nodes.

We further consider an additional scenario of heterogeneous excitabilities. Again, we are particularly interested in potential cases where considering heterogeneous excitabilities may lead to different results when compared to homogeneous excitabilities, as these may inform us on how safe is to assume that real brain networks are homogeneous with regards to excitability. If results change dramatically due to heterogeneities, then we must be careful when using the homogeneity assumption. Here we consider node excitabilities proportional to the inverse of the node degree motivated by the fact that *NI* has a strong correlation with node degree (Lopes et al., 2017). The rationale behind this choice was to observe whether by increasing the excitability of nodes with low degree led to a different distribution of *NI* compared to the homogeneous case and whether *SL* is affected in a similar way. In the case of directed networks, we study $I_{0}^{\left(i\right)}$ proportional both to node in- and out-degree separately. We fixed the range of excitabilities, such that the node with the largest number of connections had the minimum excitability $I_{0}^{\left(i\right)}$, whist the node with the smallest number of connections had the maximum $I_{0}^{\left(i\right)}$. We chose five intervals of *I*_0_: [−2.5, −0.5], [−2.5, −1.5], [−2, −1], [−1.5, −0.5], and [−5, −0.5].

## Results

### Homogeneous Excitabilities

**Figure 1:**
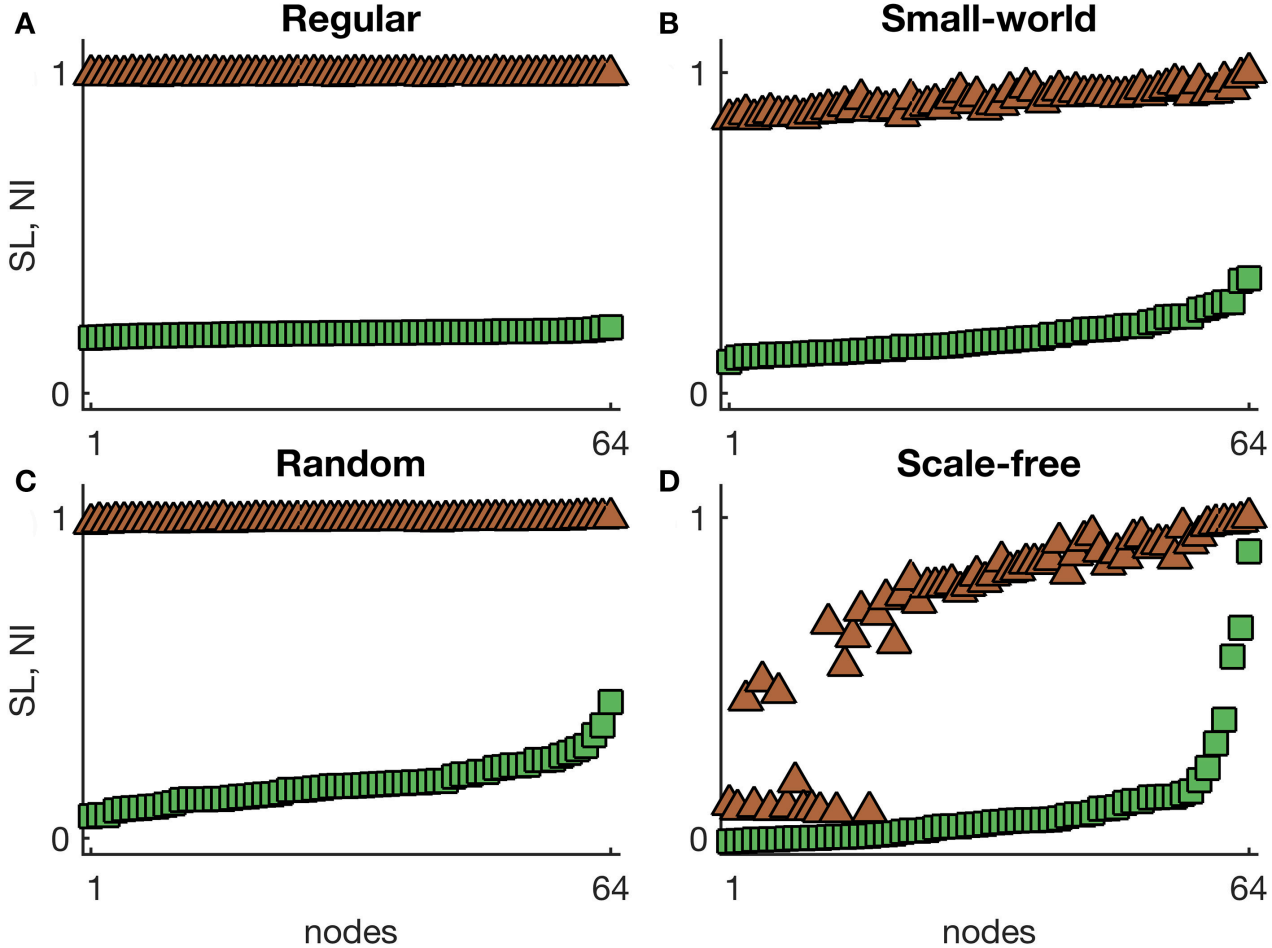
Representative *SL* and *NI* distributions of **(A)** regular, **(B)** small-world, **(C)** random, and **(D)** scale-free undirected networks with homogeneous excitabilities. The green squares correspond to *NI*^(*i*)^, whilst the brown triangles are *SL*^(*i*)^ values. The nodes were sorted such that the *NI* grows monotonously. Parameters: number of nodes *N* = 64, mean degree *c* = 4, homogeneous excitability *I*_0_ = −1.2, probability of rewiring to generate the small-world network *p* = 0.1, and degree distribution exponent of the scale-free network α = 2.6.

We first focus on the comparison of the SOZ as represented by *SL* and the effect of surgery (*NI*) in networks with homogeneous excitabilities ($I_{0}^{\left(i\right)}=I_{0}$). Figure 1 shows representative distributions of *SL* and *NI* in four different undirected network topologies. We find that *SL* and *NI* distributions are flat in regular networks (Figure 1A), which is to be expected because all nodes are equivalent. In small-world networks, we find an increased heterogeneity in both *SL* and *NI* compared to regular networks, with a high correlation between *SL* and *NI* distributions (see Figure 1B). Again, this is to be expected, because small-world networks differ slightly with respect to node degree from regular networks and node degree is highly correlated to *NI* (Lopes et al., 2017). On the other hand, these results suggest that differences in local efficiency do not significantly contribute to define *SL* and *NI*. However, random and scale-free networks present more diverging relationships between *NI* and *SL*. In random networks, whilst *SL* is uncapable of distinguishing nodes, *NI* does differentiate some nodes as being more ictogenic than others (see Figure 1C). In the case of scale-free networks, *SL* and *NI* appear to rank nodes similarly, however only a few nodes show high *NI*, while many have relatively high *SL* (see Figure 1D). This means that *NI* and *SL* are not concordant with regards to the relative importance of each node to the generation of seizures.

**Figure 2:**
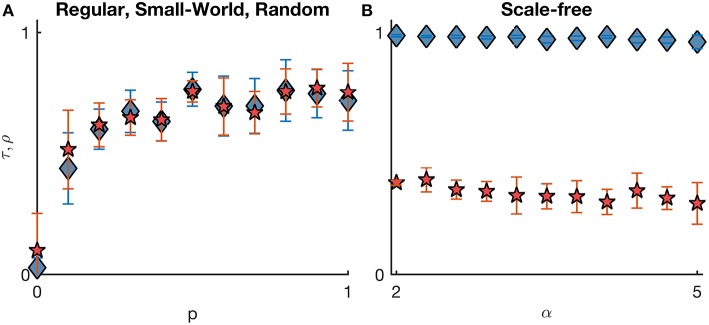
Comparison between *SL* and *NI* distributions across different undirected networks with homogeneous excitabilities. The comparison is quantified by the weighted Kendall's rank τ (blue diamonds) and Pearson correlation ρ (red pentagrams). **(A)** represents regular (*p* = 0), random (*p* = 1), and small-world networks (with varying probabilities of rewiring 0 < *p* < 1); whilst **(B)** corresponds to scale-free networks (with varying degree distribution exponent α). Error bars account for the variability of τ and ρ across 10 network realizations per each topology. All networks have mean degree *c* = 8. Other parameters are the same as in Figure 1.

To further characterize the relationship between seizure onset dynamics and underlying ictogenicity, we measured *SL* and *NI* distributions in an extensive set of networks (regular, small-world, random and scale-free networks, both directed and undirected, with different mean degrees, see Material and Methods). Figure 2 summarizes our systematic comparison of *SL* and *NI* in undirected networks (mean degree *c* = 8). τ accounts for the relation between orderings, whereas ρ is the Pearson correlation between *SL* and *NI* distributions. As observed in Figure 1A, regular networks are characterized by flat distributions of *SL* and *NI* and consequently yield low correlations between the two distributions (due to statistical fluctuations). In the case of small-world and random networks, we find a relative agreement between *SL* and *NI* distributions (ρ ≈ τ ≈ 0.68), which shows that the two measures are not interchangeable. We also observe that as we consider networks with higher rewiring probabilities *p*, the concordance between *SL* and *NI* increases, which suggests that the relationship between the two measures is enhanced in networks with lower clustering coefficients and lower average path lengths. Furthermore, Figure 2B shows that in scale-free networks the two distributions present similar orderings (τ ≈ 1) but low correlation (ρ ≈ 0.34), in line with Figure 1D. Again, this means that *SL* and *NI* are in conflict with regards to the relative importance of each node to the emergence of seizures. Figure 1D also shows that the weighted Kendall's rank and the Pearson correlation between the distributions do not change with varying the exponent α of the scale-free degree distribution. Networks with lower α have stronger hubs, i.e., subsets of nodes with higher number of connections compared to those found in networks with higher α. Our results suggest that the “strength” of the hubs do not impact on the relationship between *SL* and *NI*.

We further studied sparser and denser networks (see Figure S1). Together with Figure 2, we observe a tendency for denser networks to show lower values of τ and ρ. This is to be expected because as the networks get denser, they become more similar to regular networks and therefore their *SL* and *NI* distributions become flatter. The decrease in correlation and ordering is most noticeable in small-world and random networks given that these networks are more homogeneous than scale-free networks with respect to node degree. Also, since higher degree exponents α correspond to network more homogeneous, we find that τ and ρ also become small for sufficiently large mean degree and exponent α (see Figure S1D). Together these results indicate that the patterns observed in Figure 1 are representative of a wide range of network topologies. To further confirm these findings, we also computed *SL* and *NI* distributions in directed networks. Figure S2 demonstrates that our results are also valid in directed networks.

### Heterogeneous Excitabilities

We extend our comparison between *SL* and *NI* to networks with heterogeneous excitabilities. This is meant to represent brain tissue heterogeneities responsible for different propensities to generate seizure activity across the network. We have already seen in the previous section that network topological differences between nodes may render them individually more or less ictogenic. Now we consider nodes intrinsically different beyond network differences. This intrinsic difference is modeled as nodes being at different distances from the seizure-like state in the parameter space. As described in the Methods, we introduce two types of heterogeneous excitabilities: (1) we randomly select and attribute high excitability to a group of nodes; and (2) we define excitabilities inversely proportional to node degree.

**Figure 3:**
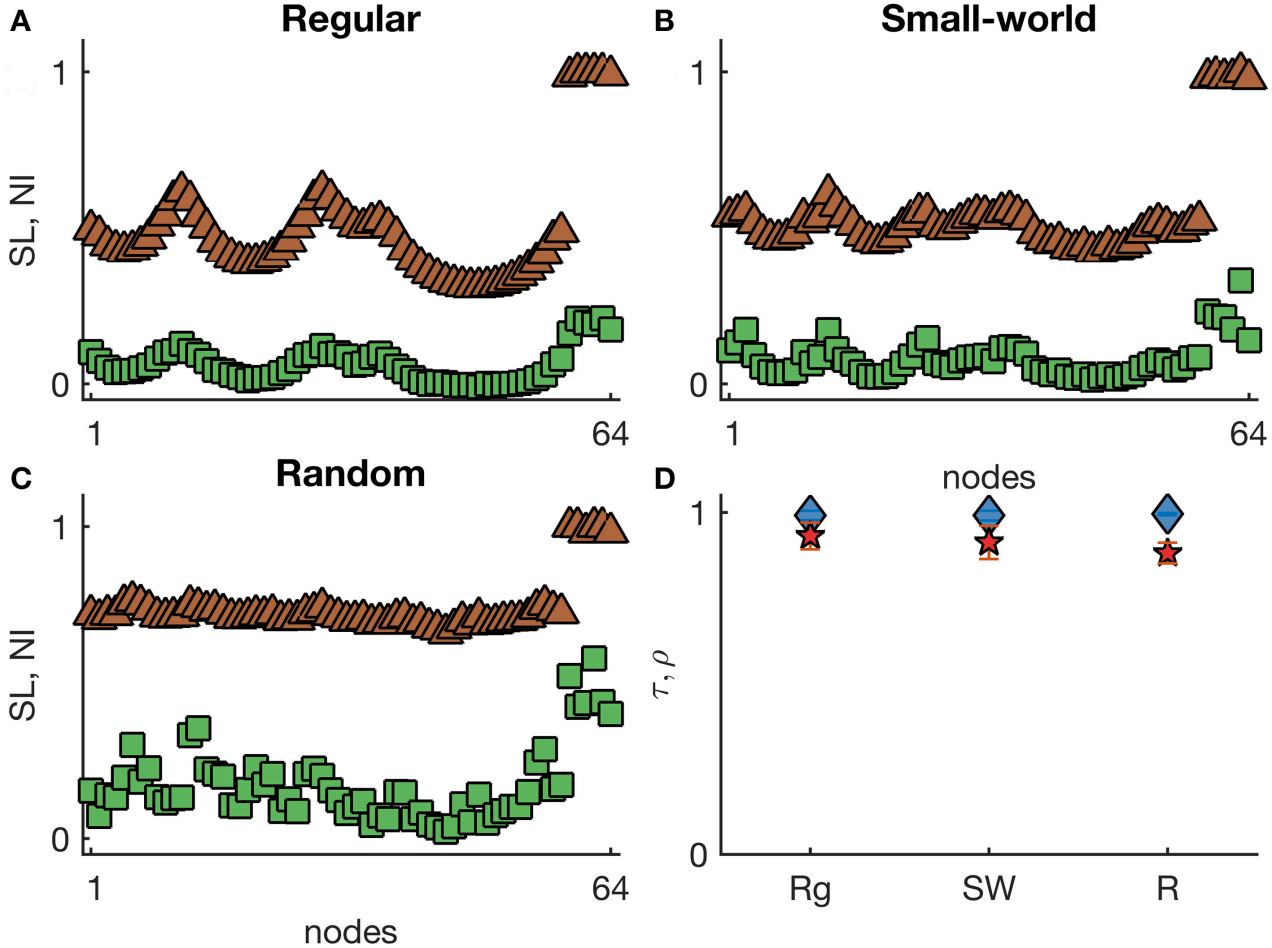
Representative *SL* and *NI* distributions of **(A)** regular, **(B)** small-world, and **(C)** random undirected networks with heterogeneous excitabilities. The green squares correspond to *NI*^(*i*)^, whilst the brown triangles are *SL*^(*i*)^ values. The nodes were sorted by their excitability $I_{0}^{\left(i\right)}$. **(D)** displays the weighted Kendall's rank τ (blue diamonds) and Pearson correlation ρ (red pentagrams) across different undirected networks with heterogeneous excitabilities (Rg: regular networks; SW: small-world networks with *p* = 0.1; and R: random networks). Node excitabilities $I_{0}^{\left(i\right)}$ were set to −1.2 apart from a group of six randomly chosen hyper-excitable nodes with $I_{0}^{\left(h\right)}=-0.1$. Error bars in **(D)** account for the variability of τ and ρ across 10 network realizations and 5 random selections of hyper-excitable nodes per each network. Other parameters are the same as in Figure 1.

Figure 3 shows representative *SL* and *NI* distributions of regular, small-world, and random networks with randomly located hyper-excitable nodes ($I_{0}^{\left(h\right)}=-0.1$). Here we find highly concordant distributions of *SL* and *NI* (τ > 0.99 and ρ > 0.88), with both measures clearly distinguishing the hyper-excitable nodes from the other nodes. These results suggest that the SOZ may be a good predictor of the EZ when brain networks are relatively homogeneous with regards to node degree and focal differences arise from intrinsic tissue heterogeneities. Figure S3A further confirms these findings in directed networks. Additionally, we also considered networks with a small fraction of nodes slightly hyper-excitable, $I_{0}^{\left(h\right)}=-1$ (note that at all other nodes *I*_0_ = −1.2). In this case, the agreement between *SL* and *NI* is similar to what we have observed in the homogeneous case (Figures 1, 2). Figure S3B displays τ and ρ in directed networks with $I_{0}^{\left(h\right)}=-1$. This implies that the heterogeneities need to be strong enough to make *SL* and *NI* distributions equivalent.

We now turn to the second case: excitabilities proportional to the inverse of node degree. This choice aims to clarify whether *SL* and *NI* are concordant when both network topology and node excitabilities are heterogeneous. Note that nodes with high degree presented higher *SL* and *NI* in Figure 1, and likewise nodes with high excitability correspond to higher *SL* and *NI* in Figure 3. Therefore, here we are studying a case where excitability and network topology “compete” in generating seizures. In other words, this scenario allows us to also observe whether network topology or node excitability are most responsible for seizure generation.

**Figure 4:**
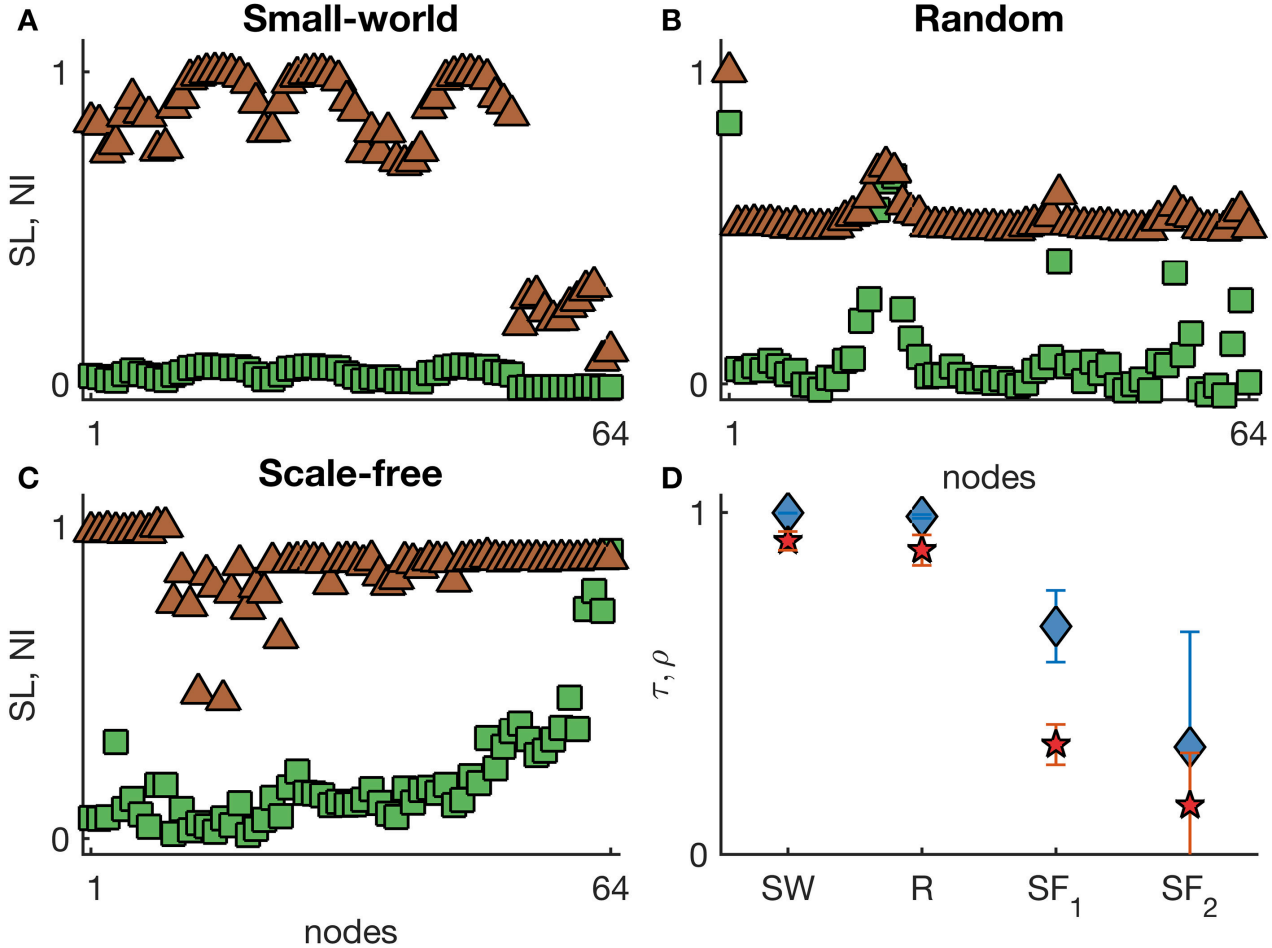
Representative *SL* and *NI* distributions of **(A)** small-world, **(B)** random, and **(C)** scale-free undirected networks with heterogeneous excitabilities. The green squares correspond to *NI*^(*i*)^, whilst the brown triangles are *SL*^(*i*)^ values. The nodes were sorted by their degree. **(D)** displays the weighted Kendall's rank τ (blue diamonds) and Pearson correlation ρ (red pentagrams) across different undirected networks with heterogeneous excitabilities (SW: small-world networks with *p* = 0.1; R: random networks; SF_1_: scale-free networks with α = 2.3; and SF_2_: scale-free networks with α = 5). Error bars account for the variability of τ and ρ across 10 network realizations per each topology. Node excitabilities $I_{0}^{\left(i\right)}$ were defined as inversely proportional to node degree within the range [−2.5, −0.5]. Other parameters are the same as in Figure 1.

Figure 4 shows representative *SL* and *NI* distributions of small-world, random, and scale-free networks with excitabilities proportional to the inverse of node degree. We observe that *SL* depends more strongly on the excitability than *NI*, particularly in scale-free networks, in which highest *NI* values correspond to nodes with highest node degree, whilst the highest *SL* is found in the nodes with lowest node degree, but highest excitability. Figure 4D demonstrates that *SL* and *NI* are mostly in agreement in small-world and random networks, whereas in scale-free networks they are different (τ < 0.67 and ρ < 0.35). These results can be further compared to the case with homogeneous excitabilities (see Figures S1A,B): we find that the heterogeneous excitabilities are responsible for a reduction in the concordance between *SL* and *NI* in scale-free networks. Note that in all these cases, we compared networks with the same mean degree so that differences could only be attributed to network topology and excitability distribution, but not to network density.

Figure S4 further supports these results in directed networks. Interestingly, in this case *SL* and *NI* are not even in agreement in directed small-world and random networks. Note that Figure S4B shows a rather high τ = 0.89 in directed scale-free networks with excitability inversely proportional to out-degree. This result is not in conflict with Figure 4D as the excitability distribution in the directed networks differs strongly from the excitability distribution in the undirected networks. We performed the same comparison using other ranges of excitability and obtained similar results (see Figure S5 for a wider range of excitability compared to Figure 4). Together these results suggest that the SOZ may be incapable of determining the EZ if network topology and node excitabilities are heterogeneous.

## Discussion

Computer models to interrogate clinical data have shown promise to quantify and predict the outcome of epilepsy surgery (Hutchings et al., 2015; Goodfellow et al., 2016; Lopes et al., 2017, 2018; Sinha et al., 2017). In this framework, clinical data was used to infer structural or functional brain networks. These networks were then analyzed using computational models of epilepsy dynamics. Potential targets for surgical resection were identified by either finding the SOZ *in silico* (Hutchings et al., 2015; Sinha et al., 2017) or modeling different possible surgeries and selecting the best based on the outcome observed in the model (Goodfellow et al., 2016; Lopes et al., 2017, 2018). Modeling the SOZ mimics the clinical approach in which it is assumed that the SOZ is a proxy of the epileptogenic zone (Rosenow and Lüders, 2001). In this study we aimed to clarify the validity of this assumption. We tested whether the SOZ, represented by *SL*, was a predictor of the effect of surgery *in silico* as measured by *NI*. We performed the comparison in different synthetic network topologies with different excitability distributions. Although in some cases *SL* is concordant with *NI*, in general the agreement is suboptimal and dependent on network topology and excitability distribution. Therefore, our results suggest caution when using the SOZ to infer targets for surgery, both in the clinical context and within computational models to predict epilepsy surgery outcome.

Our analysis was divided in two parts. First, we explored networks with homogeneous excitability distributions, i.e., all nodes were considered equivalent apart from their number of connections. We found that in sparse networks, *SL* and *NI* order nodes similarly, except in the case of scale-free networks, where their correlation is low. We have previously observed that hubs and rich-clubs have high *NI* (Lopes et al., 2017). Although, *SL* also attributes a high ranking to these nodes, the nodes do not show a high relative importance compared to other nodes. This may be a consequence of the fact that hubs can quickly spread seizure activity across the network (Stam, 2016), hence potentially masking their relative relevance for seizure generation as measured by *SL*. Thus, previous modeling approaches that used the escape time to predict surgical outcome (Hutchings et al., 2015; Sinha et al., 2017) may have achieved successful predictions due to the fact that these predictions were mostly dependent on node ranking rather than on node relative importance. However, node relative importance may be crucial in delineating the brain tissue to resect if these model-based recommendations for epilepsy surgery are to be used prospectively in the clinical setting, where it will be desirable to understand the relative impact of resecting different nodes.

In the second part of our analysis we considered networks with heterogeneous excitability distributions. Computational models of epilepsy surgery have generally assumed that network nodes are equivalent apart from network topological differences (Goodfellow et al., 2016, 2017; Khambhati et al., 2016; Lopes et al., 2017, 2018; Sinha et al., 2017). In other words, these methods have assumed that all brain regions are equally excitable, i.e., have the same intrinsic susceptibility to generate seizures and focal differences result from different connectivity patterns to other regions. Here we investigated how model predictions of targets for surgery were affected if we dropped this assumption. In particular, we examined how *SL* and *NI* compare when different network nodes had different excitabilities, i.e., nodes with different susceptibilities to be recruited into seizure dynamics. Again, the agreement between *SL* and *NI* depended on the network topology. Most notably, in scale-free networks with high excitabilities at the nodes with the lowest degree the *NI* persisted well-correlated with node degree, as in the case of homogeneous excitability distributions, whereas *SL* did not and instead was mostly determined by node excitability. These results suggest that the reliability of *SL* and consequently the reliability of the computational methods based on the seizure likelihood (Hutchings et al., 2015; Sinha et al., 2017) to predict surgical outcome are more reliant on the validity of the assumption that excitabilities are homogeneous across the network than the framework based on *NI*. These findings further suggest that node properties such as degree and betweenness centrality (Lopes et al., 2017) are significant determinants of how important a node is in terms of the overall network ictogenicity and play more of a role than intrinsic excitability. A potential consequence of this for treating seizures is that disruptions to network structure, such as those assumed to be attained in epilepsy surgery, are potentially more beneficial than targeted manipulation of local excitability. This may have implications for designing future treatments for epilepsy that can be spatially targeted, such as ablations, local drug delivery or optogenetics (Bennewitz and Saltzman, 2009; Paz and Huguenard, 2015; McGovern et al., 2016; Muller et al., 2016).

In summary, our results indicate that the SOZ may not always be a reliable proxy to infer the brain tissue to resect. The fact that seizures may emerge from one region does not imply that this region is the one responsible for the seizure emergence. Instead, our results indicate that the SOZ is not always concordant with the EZ. This understanding demands a more careful consideration of the SOZ in the clinical setting. Thus, we suggest that presurgical evaluation may be optimized if observational findings based on the SOZ are complemented with predictions based on the NI framework. In some instances, the NI framework may corroborate the hypotheses driven by the SOZ, whilst in other instances it may not, thereby advising caution. Furthermore, our results indicate that the concept of seizure likelihood should not be used *in silico* to predict epilepsy surgery outcome (Hutchings et al., 2015; Sinha et al., 2017). Predictions based on the seizure likelihood may be flawed not only because they are based on the validity of the SOZ concept, but also because they are more dependent on the assumption that node excitability may be disregarded to inform the targets for surgery. Instead, we advocate for the use of the *NI* framework which shows superior robustness with regard to the homogeneous excitability assumption, and also simulates more closely the actual surgical procedure.

Networks obtained from diffusion tensor imaging (DTI), which represent the anatomical structure of the brain's white matter, typically vary in size from 82 to 4,000 nodes (Fornito et al., 2016). Whilst we do not consider networks of this size here, we expect our results to hold for larger networks. Future studies should also examine whether *SL* and *NI* predictions obtained from such structural networks are concordant with predictions using functional networks inferred from intracranial EEG (Goodfellow et al., 2016; Lopes et al., 2017; Sinha et al., 2017). Note that DTI maps the whole brain, whereas intracranial EEG only records electrical activity from brain tissue close to the implanted electrodes. Thus, further studies should assess how these differences impact on *SL* and *NI* and their relation. Our findings may be further advanced by using more physiological detailed models (Wendling et al., 2016), namely models that describe different onset mechanisms of focal seizures (Wang et al., 2017). Finally, future investigations should study in more detail the relation between *SL*, *NI* and network topological properties. For example, it has been shown that in- and out-degree distributions have different impacts on neural dynamics (Wu et al., 2019), and therefore they may also contribute differently to *SL* and *NI*. Such studies may further advance our understanding about the relation between the SOZ and the EZ.

## Author Contributions

ML, MG, and JT contributed to the study concept and design, results interpretation, and manuscript drafting and revision. ML performed the computational simulations.

### Conflict of Interest Statement

The authors declare that the research was conducted in the absence of any commercial or financial relationships that could be construed as a potential conflict of interest.
