# Supplementary material for: A Model-Based Assessment of the Seizure Onset Zone Predictive Power to Inform the Epileptogenic Zone
Source: Front Comput Neurosci. 2019 Apr 26;13:25. doi: 10.3389/fncom.2019.00025 (PMC6498870; doi:10.3389/fncom.2019.00025)
Supplement: Supplementary file 1 [file Data_Sheet_1.PDF]

Supplementary Material

A model-based assessment of the seizure onset zone predictive power to inform the epileptogenic zone

Marinho A. Lopes*, Mark P. Richardson, Eugenio Abela, Christian Rummel, Kaspar Schindler, Marc Goodfellow, John R. Terry

*** Correspondence:** Marinho A. Lopes: m.lopes@exeter.ac.uk

1 Supplementary Figures

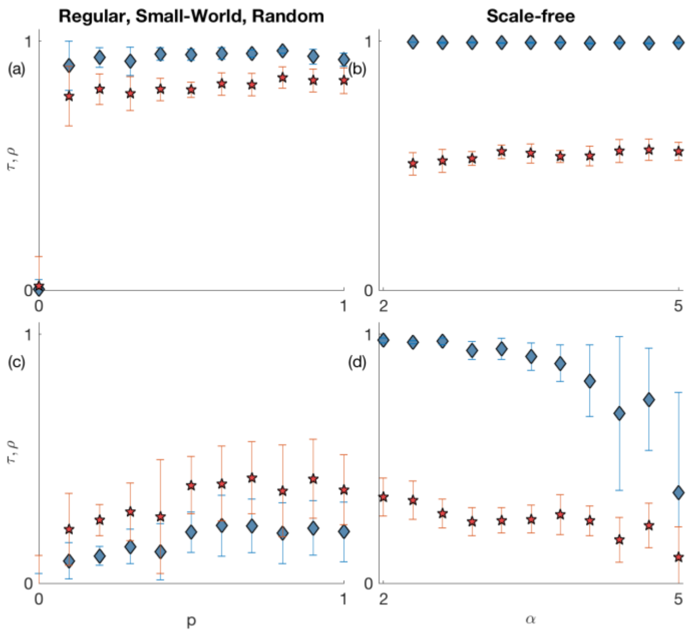

Supplementary Figure 1. Comparison between SL and NI distributions across different undirected networks with homogeneous excitabilities. The comparison is quantified by the weighted Kendall's rank τ (blue diamonds) and Pearson correlation ρ (red pentagrams). The left column (a,c) represents regular ($p = 0$), random ($p = 1$), and small-world networks (with varying probabilities of rewiring $0 < p < 1$); whilst the right column (b,d) corresponds to scale-free networks (with varying degree distribution exponent α). Each row describes topologies with different mean degrees: (a-b) $c = 4$, and (c-d) $c = 16$. Error bars account for the variability of τ and ρ across 10 network realizations per each topology. Other parameters are the same as in Figure 1.

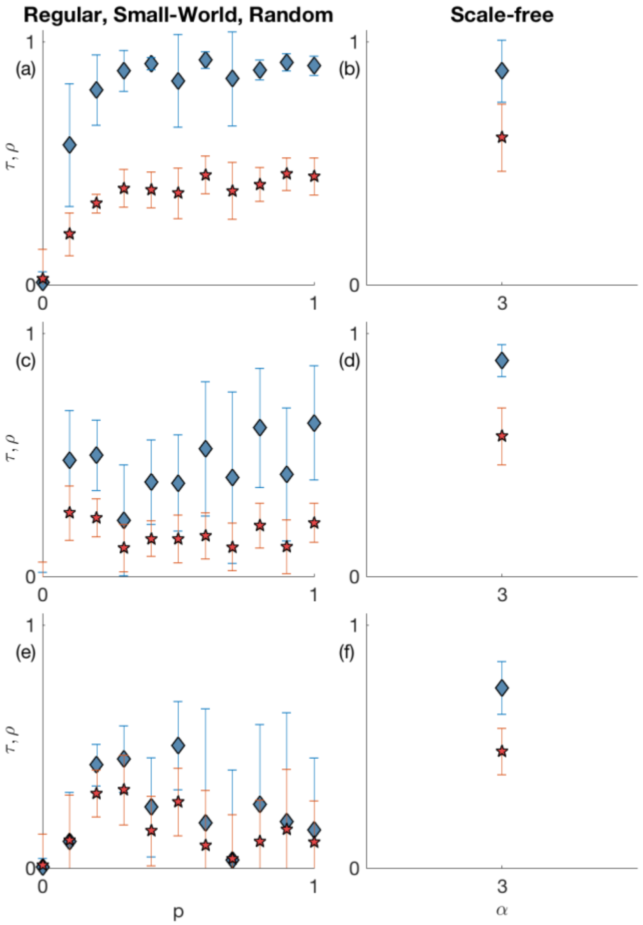

Supplementary Figure 2. Comparison between SL and NI distributions across different directed networks with homogeneous excitabilities. The comparison is quantified by the weighted Kendall's rank τ (blue diamonds) and Pearson correlation ρ (red pentagrams). The left column (a,c,e) represents regular ($p = 0$), random ($p = 1$), and small-world networks (with varying probabilities of rewiring $0 < p < 1$); whilst the right column (b,d,f) corresponds to scale-free networks. Each row describes topologies with different mean degrees: (a-b) $c = 4$, (c-d) $c = 8$, and (e-f) $c = 16$. Error bars account for the variability of τ and ρ across 10 network realizations per each topology. Other parameters are the same as in Figure 1.

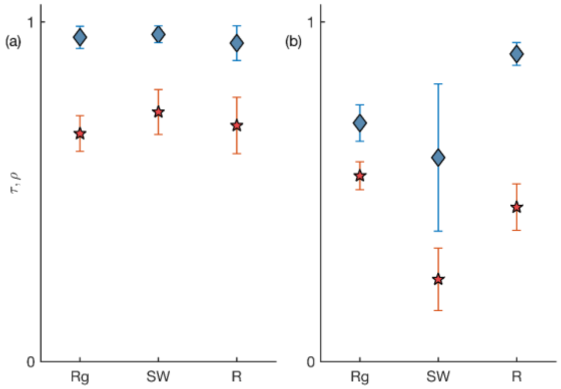

Supplementary Figure 3. Comparison between SL and NI distributions across different directed networks with heterogeneous excitabilities. The comparison is quantified by the weighted Kendall's rank τ (blue diamonds) and Pearson correlation ρ (red pentagrams) across different directed networks (Rg: regular networks; SW: small-world networks with $p = 0.1$; and R: random networks). Node excitabilities $I_0^{(i)}$ were set to -1.2 apart from a group of six randomly chosen hyper-excitable nodes with (a) $I_0^{(h)} = -0.1$, and (b) $I_0^{(h)} = -1$. Error bars account for the variability of τ and ρ across 10 network realizations and 5 random selections of hyper-excitable nodes per each network. Other parameters are the same as in Figure 1.

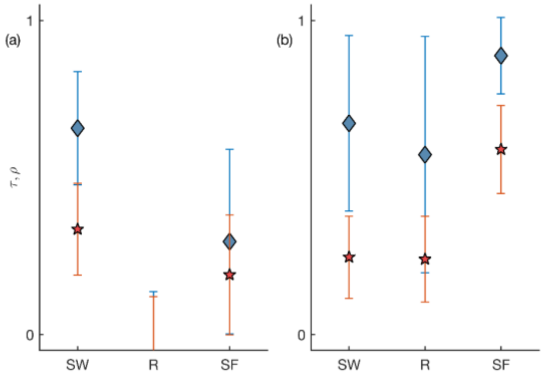

Supplementary Figure 4. Comparison between SL and NI distributions across different directed networks with heterogeneous excitabilities. The comparison is quantified by the weighted Kendall's rank τ (blue diamonds) and Pearson correlation ρ (red pentagrams) across different directed networks (SW: small-world networks with $p = 0.1$; R: random networks; and SF: scale-free networks). Error bars account for the variability of τ and ρ across 10 network realizations per each topology. Node excitabilities $I_0^{(i)}$ were defined as inversely proportional to node (a) in-degree and (b) out-degree within the range $[-2.5, -0.5]$. Other parameters are the same as in Figure 1.

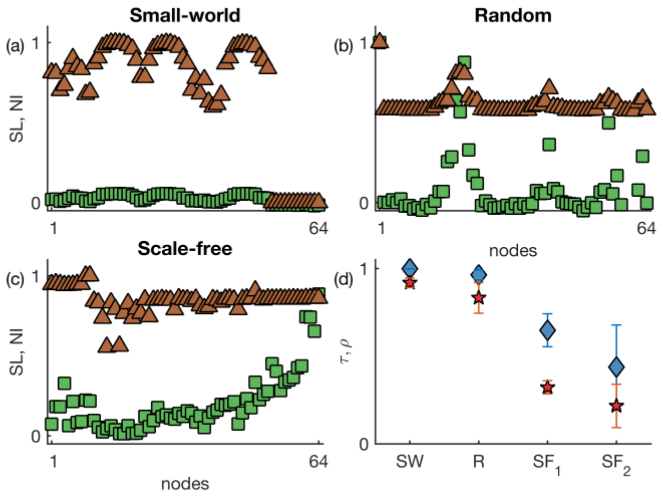

Supplementary Figure 5. Representative SL and NI distributions of (a) small-world, (b) random, and (c) scale-free undirected networks with heterogeneous excitabilities. The green squares correspond to $NI^{(i)}$, whilst the brown triangles are $SL^{(i)}$ values. The nodes were sorted by their degree. Panel (d) displays the weighted Kendall's rank τ (blue diamonds) and Pearson correlation ρ (red pentagrams) across different undirected networks with heterogeneous excitabilities (SW: small-world networks with $p = 0.1$; R: random networks; SF_1: scale-free networks with $\alpha = 2.3$; and SF_2: scale-free networks with $\alpha = 5$). Error bars account for the variability of τ and ρ across 10 network realizations per each topology. Node excitabilities $I_0^{(i)}$ were defined as inversely proportional to node degree within the range $[-5, -0.5]$. Other parameters are the same as in Figure 1.
